# The complete mitochondrial genome of *Reticulitermes ovatilabrum* (Isoptera: Rhinotermitidae)

**DOI:** 10.1080/23802359.2021.1882903

**Published:** 2021-03-11

**Authors:** Tong Chen, Jiaojun Qian, Qingfeng Dai, Chengyuan Pan, Baoling Li, Dayu Zhang

**Affiliations:** a College of Agricultural and Food Science, Zhejiang A&F University, Hangzhou, PR China; b National Termite Control Center, Hangzhou, PR China

**Keywords:** *Reticulitermes ovatilabrum*, mitochondrial genome

## Abstract

The mitochondrial genome of the *Reticulitermes ovatilabrum* is 15,913 bp in length and encodes 37 genes including 13 protein-coding genes (PCGs), 22 transfer RNA genes (tRNA), 2 ribosomal RNA genes (rRNA), and a non-coding control region (D-loop). The percentage of A/T (65.59%) is much higher than that of C/G (34.41%). The phylogenetic tree revealed that *R. ovatilabrum* was closest to *R. kanmonensi* and *R. periflaviceps*. The mitochondrial genome of the *R. ovatilabrum* provides a resource for evolutional analysis within termites especially *Reticulitermes*.

*Reticulitermes ovatilabrum* (Isoptera: Rhinotermitidae) was first reported in Guangxi, China (Xia and Fan 1981; Huang et al. 2000), which has obvious features of *Reticulitermes*. *R. ovatilabrum* also contains unique oval labrum which is different from other *Reticultitermes* species. Here, this study provides the complete mitochondrial genome sequence of *R. ovatilabrum*.

Specimens were collected from Niutoushan Mountain (East Longitude 119°46′ and North Latitude 28°64′), Zhejiang, China in 2019 and kept in the Insect Lab at Zhejiang A&F University, China (accession number NTS-05). Total genomic DNA was extracted from the heads of 10 worker termites using the Insect DNAKit following the manufacturer’s instructions (Sangon Biotech, Shanghai, China). Fifteen primer pairs were designed to amplify the complete mitogenome by PCR and then sequenced using Sanger sequencing technology. DNA sequences were assembled by Seqman program from DNASTAR, Madison, WI (Lasergene version 7.1). MITOS Web Server was utilized for mitogenome annotation and predicted secondary structure of transfer RNAs (Bernt et al. 2013).

The complete mitochondrial genome sequence of *R. ovatilabrum* is 15,913 bp in length (GenBank: MW015945). It encodes 13 protein-coding genes (PCGs), 22 transfer RNA (tRNA) genes, 2 ribosomal RNA (rRNA) genes, and a non-coding control region (D-loop). A total of 14 genes are transcribed on the L-strand (*nad1, nad4, nad4L, nad5, trnQ*, *trnC*, *trnY*, *trnF*, *trnH*, *trnP*, *trnL1*, *trnV*, *rrnS*, and *rrnL*) and other genes are located on the H-strand. The base composition of *R. ovatilabrum* mitochondrial genome has a high percentage of A/T (65.59%), which was similar to other *Reticulitermes* (Cameron and Whiting 2007; Liu et al. 2019; Ye et al. 2019). Furthermore, there are 18 intergenic spacers and 8 overlapping regions in *R. ovatilabrum* mitochondrial genome. The intergenic spacers contain 123 bp and overlapping regions have 47 bp in total, respectively.

PCGs have a total of 11,174 bp in length. The start codons of all PCGs are ATN. Except for *nad1* with TAG, *cox2* and *nad5* with incomplete T as stop codon, the other protein genes all adopt TAA as stop codon. Except *trnS1* which lacks the dihydrorubamide (DHU) arm, the remaining 21 tRNAs have typical clover-leaf structures. The 16S rRNA (*rrnL*) and 12S rRNA (*rrnS*) gene contains 1327 and 737 bp, respectively. The control region (D-loop) with 1103 bp is located between *rrnS* and *trnI* with a high A/T content（68.36%).

The maximum likelihood method based on the all PCG nucleotide sequences was used to analyze the phylogeny of *Reticulitermes* containing *R. ovtilabrum* (Guindon et al. 2010). *Coptotermes formosanus*as (Isoptera: Rhinotermitidae) and *Mastotermes darwiniensis* (Isoptera: Mastotermitidae) were chosen as the outer groups for phylogenetic analysis. *R. ovatilabrum* constituted a sister group to *R. kanmonensis* and *R. periflaviceps* (Figure 1).

**Figure 1.**
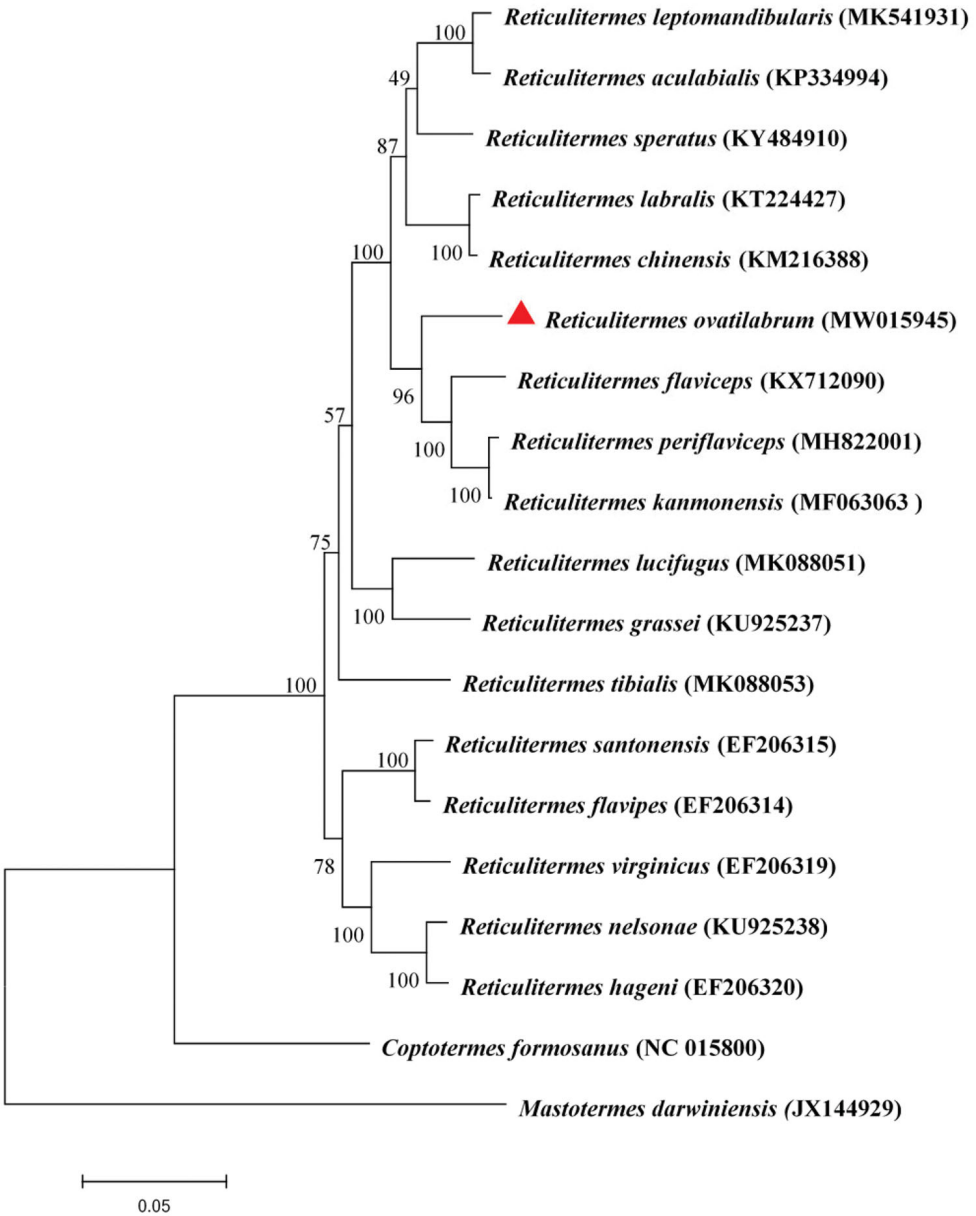
Maximum Likelihood phylogenetic tree of mitogenomes of *Reticulitermes* (bootstrap values based on 1000 replicates). *Coptotermes formosanusas* and *Mastotermes darwiniensis* were used as outgroups. Branch names were presented as species names and GenBank accession number.

## Data Availability

The genome sequence data that support the findings of his study are openly available in GenBank of NCBI at https://www.ncbi.nlm.nih.gov/ under the accession number MW015945. The associated BioProject, Bio-Sample and SRA numbers are PRJNA681300, SAMN16948895, SRR13170425-SRR13170439 respectively.
